# Is CPAP the Key to Reduce the Cardiovascular Risk in Patients with OSA?

**DOI:** 10.1055/s-0045-1801855

**Published:** 2025-08-07

**Authors:** Kiran Abraham-Aggarwal, Ashutosh Kacker

**Affiliations:** 1 School of Industrial and Labor Relations, Cornell University, Ithaca, NY, United States; 2 Department of Otorhinolaryngology — Head and Neck Surgery, Joan and Sanford I. Weill Medical College, Cornell University, Ithaca, NY, United States

**Keywords:** otolaryngology, sleep apnea, cardiovascular disease, sleep medicine specialty, CPAP

## Abstract

**Introduction** Obstructive sleep apnea (OSA) is a prevalent condition often managed with continuous positive airway pressure (CPAP) treatment. There is an ongoing debate about the link between OSA and cardiovascular disease (CVD), but the existing evidence points toward an association.

**Objective** The present study aims to investigate whether CPAP is an effective strategy to reduce the risk of developing CVD risk among OSA patients.

**Methods** The literature search was conducted in collaboration with Weill Cornell Medical College librarians, and we identified the five most recent papers highlighting the correlation between CPAP treatment for OSA and CVD risk reduction.

**Results** Recent studies highlight the link between OSA and CVD, emphasizing the potential of the CPAP therapy to reduce the risks of developing CVD and the mortality associated with the condition. The analyses by Guo et al., Peker et al., Khan et al., and Wickwire et al., collectively involving thousands of patients across various study designs, including randomized clinical trials and retrospective cohort studies, consistently demonstrate that the CPAP treatment improves cardiovascular outcomes by lowering blood pressure, reducing sleepiness scores, and significantly decreasing the risk of serious CVD events, especially with consistent adherence to therapy.

**Conclusion** Although further studies are needed to confirm these findings among patients with consistent high compliance to the CPAP treatment, it remains reasonable to continue to prescribe CPAP as the first-line therapy for OSA due to its potential cardiovascular benefits and low associated risks.

## Question

Is CPAP the key to reduce the CV risk in OSA patients?

## Introduction

Obstructive sleep apnea (OSA) is a common condition among the general population, and the CPAP treatment is the standard first-line therapy. There has been significant debate over whether OSA increases the predisposition to develop cardiovascular disease (CVD); however, though the data are conflicting, most physicians would recommend the treatment of OSA to reduce CVD risk. In the present paper, we aim to examine if the CPAP treatment is the best practice to reduce the risk of developing CVD in OSA patients.

## Literature Review

In 2023, Labarca et al. 1 published an extensive study on the incidence of cardiovascular (CV) morbidity and mortality among patients who experience the physiological burdens of OSA, which include hypoxic burden (HB), ventilatory burden (VB), ventilatory deficit, and arousal burden. The authors used the Multi-Ethnic Study of Atherosclerosis (MESA) and the Osteoporotic Fractures in Men (MrOS) study to quantify the impact of OSA on the CV risk. These studies had a cumulative sample size of 4,600 patients. In the primary analysis, the MESA study showed that every standard deviation increase in the HB led to an increase of 45% in the risk of incident CVD events, and, in the MrOS study, there was a 13% increased risk. Further, for the VB, in the MESA study, there was a 35% increased risk, and, in the MrOS study, 12%. Though the VB can predict the CVD risk in OSA patients, the MESA and MrOS studies show that the HB more strongly correlates to the CVD risk. Labarca et al. 1 demonstrated that the HB is a strong predictor of increased CVD risk in OSA patients.

In 2016, Guo et al. 2 conducted a meta-analysis which included 18 randomized clinical trials (RCTs), with a total sample size of 4,146 patients. They evaluated the risk of CVD events and mortality among OSA patients treated with CPAP. Additionally, they assessed the effect of CPAP on the Epworth Sleepiness Score (ESS) and blood pressure (BP), both of which may contribute to CVD. The CPAP treatment significantly decreased the ESS. Regarding BP, the CPAP therapy significantly lowered both systolic and diastolic BP compared with the control groups. There was a significant decrease in the daytime and nocturnal diastolic BP and a decrease in the nocturnal systolic BP. Additionally, Guo et al. 2 found that CPAP had a mild beneficial effect on CV events in patients who used it for ≥ 4 hours per night and, with longer treatment, it also decreased CV mortality to levels similar to those of patients without OSA. Overall, this meta-analysis suggests that CPAP is a safe and effective treatment for OSA patients, and that the CPAP therapy is associated with a trend of decreased risk of CVD events.

In 2016, Peker et al. 3 conducted an RCT with 244 patients to investigate the relationship between OSA and coronary artery disease (CAD), is a common subtype of CVD with a high mortality risk. Obstructive sleep apnea is also a common condition in patients with CAD, but it often goes undiagnosed. The primary endpoint in this trial was the first event of repeat revascularization, myocardial infarction, stroke, or CV mortality. In the on-treatment analysis, there was a significant difference between the groups in the incidence rates based on CPAP use for ≥ 4 hours per night. Their findings suggest that CPAP use for > 4 hours per night was associated with a reduced risk of the composite endpoint in patients with CAD and OSA after adjustment for baseline comorbidities and compliance with the treatment.

In 2018, Khan et al. 4 conducted a meta-analysis on the relationship between OSA and CV events and the efficacy of CPAP therapy in alleviating OSA. The authors first looked at the Sleep Apnea Cardiovascular Endpoints (SAVE) trial, an RCT which aimed to determine whether the CPAP treatment for OSA could reduce the risk of presenting serious CV events. In its original findings, the SAVE trial reported that the CPAP therapy did not significantly reduce the risk of presenting CV events. However, upon further analysis, Khan et al. 4 adjusted for better compliance time (≥ 4 hours per night) and ultimately found a non-significant trend favoring the CPAP therapy. The results of the meta-analysis showed that, though further randomized trials are required, a significant risk reduction in major adverse CV events was observed in the sensitivity analyses excluding two studies with low CPAP adherence time per night and the results from the SAVE trial.

In 2021, Wickwire et al. 5 performed a retrospective study to further understand the efficacy of the CPAP treatment in preventing CV events in OSA patients. They used a 5% random sample of Medicare administrative records from 2008 to 2015, generating a population of 5,024 Medicare beneficiaries, with an average age of 72 years. The cohort consisted of individuals with OSA submitted to at least 1 session of the CPAP treatment within 6 months of the diagnosis. Additionally, the cohort presented a high prevalence of CVD and CVD morbidities. Wickwire et al. 5 determined that adherence to the CPAP treatment significantly reduced the risk of presenting new CVD events. Further, the authors found that the CPAP treatment reduced the incidence of new CVD events over 25 months. Subjects undergoing ≥ 4 hours per night of CPAP presented a more significant decrease in new CVD events compared to individuals who underwent < 4 hours per night of CPAP. This study sheds light on the potential advantages of consistent CPAP therapy in lowering the chances of experiencing CV events among the elderly.

## Conclusion

Currently, no RCTs conclusively demonstrate that CPAP reduces the incidence of CV. While the data suggest that CPAP may lower the CV risk in OSA patients, this benefit has been observed primarily in subgroup analyses. The CPAP treatment has been shown to reduce BP and improve sleepiness, both of which are pathways that could potentially reduce the CV risk. Therefore, with high CPAP compliance, there is a possibility of reducing the CVD risk. Although further studies are needed to confirm these findings among with consistent high compliance to the CPAP treatment, it remains reasonable to continue to prescribe CPAP as the first-line therapy for OSA due to its potential CV benefits and low associated risks.

## Level of Evidence

Studies regarding OSA and the treatment for OSA to reduce CVD risk vary in terms of the level of evidence. The present literature review focuses on 5 studies, which are summarized in Table 1 . Three of the studies 2 4 5 have level 2 of evidence, and two 1 3 have level 1.

**Table 1:** Summary of the studies on the efficacy of the CPAP therapy

**Study title**	**Level of evidence**	**Summary**	**Sample size**
Sleep apnea physiological burdens and cardiovascular morbidity and mortality.	Level 1	Found that the hypoxic burden is a strong predictor of increased cardiovascular disease risk in OSA patients.	4,600 patients
Effects of CPAP therapy on cardiovascular events and mortality in patients with OSA: a meta-analysis.	Level 2	Highlights the positive impact of the CPAP treatment on OSA patients, revealing significant improvements in sleepiness levels and blood pressure, and suggests a potential decrease in CV events associated with longer-term CPAP use.	4,146 patients
Effect of positive airway pressure on cardiovascular outcomes in coronary artery disease patients with nonsleepy obstructive sleep apnea. The RICCADSA randomized controlled trial.	Level 1	Consistent use of CPAP for > 4 hours per night is associated with a lower incidence of adverse CV outcomes in individuals with CAD and undiagnosed OSA.	244 patients
A meta-analysis of continuous positive airway pressure therapy in prevention of cardiovascular events in patients with obstructive sleep apnoea.	Level 2	Revealed a potential trend favoring CPAP therapy in reducing major adverse CV events in OSA patients when compliance time was adjusted and certain studies were excluded.	4,268 patients
CPAP adherence reduces cardiovascular risk among older adults with obstructive sleep apnea.	Level 2	Adherence to the CPAP treatment significantly reduced the risk of new CV events, highlighting the potential benefits of consistent CPAP therapy in the elderly population.	5,024 patients

Abbreviations: CAD, coronary artery disease; CPAP continuous positive airway pressure; CV, cardiovascular; OSA, obstructive sleep apnea.

## References

[1] Labarca G Vena D Hu W-H Sleep apnea physiological burdens and cardiovascular morbidity and mortality Am J Respir Crit Care Med 2023 208 07 802 813 10.1164/rccm.202209-1808oc 37418748 PMC10563185

[2] Guo J Sun Y Xue L-J Effect of CPAP therapy on cardiovascular events and mortality in patients with obstructive sleep apnea: a meta-analysis Sleep Breath 2016 20 03 965 974 10.1007/s11325-016-1319-y 26873722

[3] Peker Y Glantz H Eulenburg C Wegscheider K Herlitz J Thunström E Effect of positive airway pressure on cardiovascular outcomes in coronary artery disease patients with nonsleepy obstructive sleep apnea. the RICCADSA randomized controlled trial Am J Respir Crit Care Med 2016 194 05 613 620 10.1164/rccm.201601-0088oc 26914592

[4] Khan S U Duran C A Rahman H Lekkala M Saleem M A Kaluski E A meta-analysis of continuous positive airway pressure therapy in prevention of cardiovascular events in patients with obstructive sleep apnoea Eur Heart J 2018 39 24 2291 2297 10.1093/eurheartj/ehx597 29069399

[5] Wickwire E M Bailey M D Somers V K CPAP adherence reduces cardiovascular risk among older adults with obstructive sleep apnea Sleep Breath 2021 25 03 1343 1350 10.1007/s11325-020-02239-2 33141315

